# Diabetes mellitus is associated with increased prevalence and severity of COMISA: evidence from the nationwide TURKAPNE cohort

**DOI:** 10.1007/s44470-026-00109-4

**Published:** 2026-07-02

**Authors:** Canan Gündüz Gürkan, Aylin Pihtili, Esen Kiyan, Mehmet Sezai Tasbakan, Özen K. Basoglu, Semih Arbatli, Senay Aydin, Aykut Cilli, Neşe Dursunoglu, Burcu Baran, Yüksel Peker, Turkapne Study Group, Ahmet Ursavaş, Ali Nihat Annakkaya, Alperen Aksakal, Aydın Balcı, Ayfer Utkusavaş, Aylin Özsancak Ugurlu, Ayşe Deniz Elmalı, Baran Balcan, Başak Gönen, Bilkay Serez Kaya, Burcu Oktay Arslan, Caner Çınar, Demet İlhan Algın, Derya Karadeniz, Dilara Mermi Dibek, Dursun Dursunoglu, Duygu Özol, Ebru Çakır Edis, Ebru Duygu, Ege Güleç Balbay, Ersin Günay, Fuat Erel, Funda Aksu, Gökhan Kırbaş, Gülçin Benbir Şenel, Gülgün Çetintaş Afşar, Gülin Sünter, Hadice Selimoğlu Şen, Hamza Ogun, Hasan Akça, Hikmet Firat, Hilal Türkmen Kaya, Işıl Yazıcı Gençdal, İbrahim Öztura, İlker Yılmam, Kadriye Ağan, Mehmet Ali Habeşoğlu, Mehmet Karadağ, Melike Banu Yüceege, Metin Akgün, Muhammed Emin Akkoyunlu, Mustafa Saygın, Nejat Altintas, Nur Aleyna Yetkin, Nurhan Sarıoğlu, Oğuz Osman Erdinç, Onur Bulut, Oya İtil, Oya Öztürk, Önder Öztürk, Özge Aydın Güçlü, Özlem Erçen Diken, Pınar Bekdik Şirinocak, Pınar Yıldız Gülhan, Sadık Ardıç, Selma Fırat, Sema Saraç, Sevgi Ferik, Sezgi Şahin Duyar, Sinem Berik Safçi, Sinem N. Sökücü, Süreyya Yılmaz, Tunahan Anber, Uluğ Bey Hayri, Ülkü Dübüş Hoş, Ümmühan Okur, Vasfiye Kabeloğlu, Yeliz Celik, Zahide Yılmaz, Zeynep Zeren Uçar

**Affiliations:** 1 Department of Pulmonary Medicine, Süreyyapasa Chest Diseases Research and Training Hospital, Istanbul, Türkiye; 2 Department of Pulmonary Medicine, Istanbul University School of Medicine, Istanbul, Türkiye; 3 Department of Pulmonary Medicine, Ege University School of Medicine, Izmir, Türkiye; 4 Koc University Research Center for Translational Medicine (KUTTAM), Koc University School of Medicine, Davutpasa Cad No 4, Zeytinburnu, Istanbul, TR 34010 Türkiye; 5 Department of Neurology, Yedikule Chest Diseases and Thoracic Surgery Education and Research Hospital, Istanbul, Türkiye; 6 Department of Pulmonary Medicine, Akdeniz University School of Medicine, Antalya, Türkiye; 7 Department of Pulmonary Medicine, Pamukkale University School of Medicine, Denizli, Türkiye; 8 Department of Pulmonary Medicine, Erciyes University School of Medicine, Kayseri, Türkiye; 9 Department of Pulmonary Medicine, Koc University School of Medicine, Istanbul, Türkiye; 10 Department of Molecular and Clinical Medicine, Institute of Medicine, University of Gothenburg, Sahlgrenska Academy, Gothenburg, Sweden; 11 Department of Clinical Sciences, Respiratory Medicine and Allergology, Lund University School of Medicine, Lund, Sweden; 12 Division of Pulmonary, Allergy, and Critical Care Medicine, University of Pittsburgh School of Medicine, Pittsburgh, PA USA

**Keywords:** Insomnia, Sleep apnea, Comisa, Polysomnography, Diabetes mellitus

## Abstract

**Background:**

Comorbid insomnia and obstructive sleep apnea (COMISA) is increasingly recognized as a clinically important sleep disorder phenotype with potential cardiometabolic consequences. However, its association with diabetes mellitus (DM) remains insufficiently characterized.

**Methods:**

This cross-sectional analysis included 12,715 adults with suspected obstructive sleep apnea (OSA) from the nationwide multicenter TURKAPNE cohort who underwent full-night polysomnography. Insomnia symptoms were assessed using standardized questionnaire items, and OSA was defined as apnea–hypopnea index (AHI) ≥ 5 events/hour. COMISA was defined as the coexistence of insomnia symptoms and OSA. DM was identified based on self-reported physician diagnosis or use of antidiabetic medications. Multivariable logistic regression was performed to evaluate independent variables associated with COMISA.

**Results:**

COMISA was present in 3,275 patients (25.7%). DM was significantly more prevalent among patients with COMISA compared with those without COMISA (22.8% vs. 15.4%, *p* < 0.001). Overall, COMISA was observed in 33.9% of diabetic individuals and 24.1% of non-diabetic individuals. In multivariable analysis adjusted for age, sex, body-mass-index, smoking status, education level, and comorbidities, DM remained independently associated with COMISA (adjusted OR 1.17; 95% CI 1.05–1.31; *p* = 0.006). Among COMISA patients, those with DM exhibited greater sleep fragmentation and more severe sleep-disordered breathing, including higher AHI (30.0 vs. 24.0 events/h) and oxygen desaturation index (26.0 vs. 20.0 events/h) (*p* < 0.001).

**Conclusions:**

DM is independently associated with COMISA and with more severe sleep and respiratory disturbances within this phenotype. These findings highlight the need for integrated screening of sleep disorders in individuals with DM.

**Trial registration:**

ClinicalTrials gov (NCT02784977).

**Graphical abstract:**

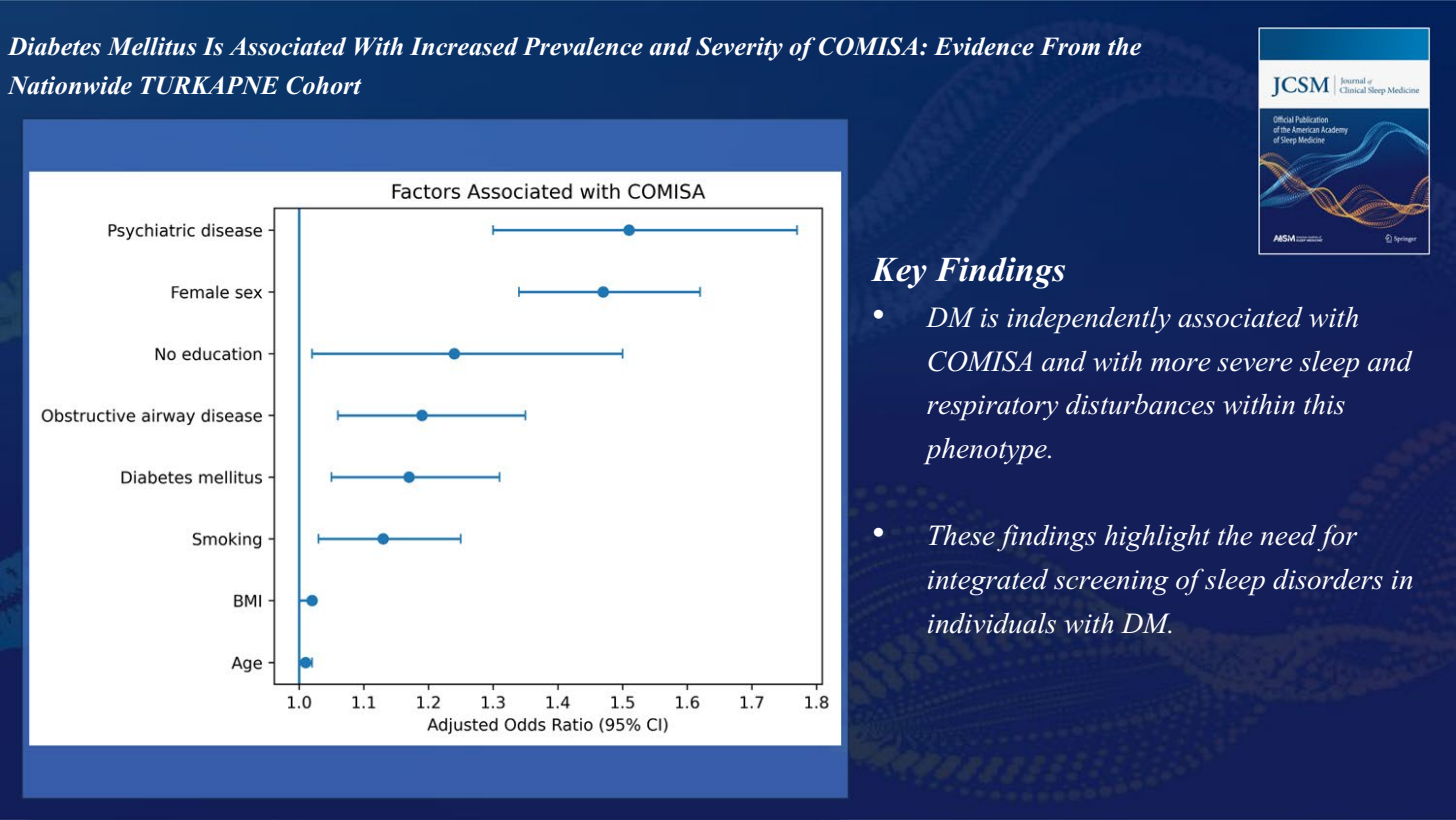

## Introduction

Obstructive sleep apnea (OSA) is defined by repetitive partial or complete obstruction of the upper airway during sleep, leading to intermittent hypoxemia and sleep fragmentation. These core pathological features are likely to play a role in the causal pathway leading to metabolic dysfunction [1, 2]. The prevalence of OSA is estimated to be approximately 60% in the diabetic population, significantly higher than in the general population (9%–38%) [3]. In the bidirectional association between OSA and diabetes mellitus (DM), the underlying pathways that link OSA to insulin resistance and type 2 DM include enhanced sympathetic activity, promoted oxidative stress and systemic inflammation [4, 5].

Insomnia is a sleep disorder characterized by frequent difficulties in initiating or maintaining sleep, along with associated daytime impairment, and evidence support that it has adverse effects on cardiometabolic diseases [6, 7]. Insomnia is also defined as a prevalent sleep disorder in people with type 2 DM, affecting approximately 39% of this population compared with the general population [8].

The co-occurrence of OSA and insomnia (COMISA) is remarkably common, affecting approximately 30–50% of OSA patients and 30–40% of chronic insomnia patients [9]. Growing evidence suggests that COMISA drives higher risk for adverse outcomes, including incident DM and cardiovascular disease, than either disorder alone, particularly when characterized by objective measures of physiological burden [10]. In patients with type 2 DM, COMISA demonstrates a potent negative synergistic effect on health outcomes [11].

Despite independent associations between type 2 DM and OSA and insomnia have been demonstrated in the literature, there is limited knowledge regarding the potential effects of COMISA on type 2 DM. Accordingly, this study aimed to investigate the associations between COMISA and DM, and to characterize differences in sleep architecture and respiratory parameters according to diabetes status in a large multicenter cohort.

## Material and methods

### Methods

The national multicenter Turkish Sleep Apnea Database (TURKAPNE) prospective cohort has been described previously [12]. Patients with suspected OSA and aged between 18–80 years from 34 centers have been registered in the study since 2017. Analysis included demographic data, anthropometric measurements, comorbidities, questionnaires, and polysomnography (PSG) data. Patients with limited life expectancy due to end stage renal disease or terminal stage malignancies, alcohol addiction, and the use of mandibular advancement devices or positive airway pressure (PAP) therapy were excluded.

### Ethical considerations

This study was performed in accordance with the principles of the 1975 Declaration of Helsinki and was approved by the Ethics Committee of Marmara University Faculty of Medicine, Istanbul (approval number: 09.2016.311). Written informed consent was obtained from all participants prior to enrollment. The trial was registered at ClinicalTrials.gov (NCT02784977).

### Data management and quality control

The TURKAPNE registry operates through a dedicated web-based system that transfers data to a centralized database (www.turkapne.org) hosted by Hetzner Online GmbH in Jestetten, Germany [12]. Each participating center is provided with unique login credentials to access a clinical reporting platform structured into predefined modules to ensure standardized data entry. Centers are able to access and manage only the records of patients enrolled at their own site. All participants are registered using coded identifiers, while personally identifiable information is securely maintained at the respective reporting center in a written log. Data integrity is overseen by an independent monitoring board that performs random audits and has full read access to the entire database.

### Demographic and anthropometric characteristics, comorbidities, and medications

Baseline demographic and lifestyle information was systematically collected, including age, sex, marital status, educational level, driver’s license possession, and habits related to smoking and alcohol consumption. Anthropometric assessments encompassed height, weight, body mass index (BMI), heart rate, and blood pressure. Comorbid conditions, such as obstructive airway disease (chronic obstructive pulmonary disease and asthma), were documented based on both physician diagnosis and participant self-report. Additionally, current medication use was recorded, and for female participants, menstrual or menopausal status was noted.

### Excessive daytime sleepiness

The Turkish version of the Epworth Sleepiness Scale (ESS) was used in the assessment of excessive daytime sleepiness [13]. The Epworth Sleepiness Scale is an eight-item self-report questionnaire, with each item scored from 0 (no chance of dozing) to 3 (high chance of dozing); total scores range from 0 to 24, and a score ≥ 11 denotes clinically significant excessive daytime sleepiness [14].

### Polysomnography

Overnight in-laboratory PSG was performed in all patients. Coffee, alcohol, and sedative medications prohibited on the day of the study. Electroencephalography, electromyography, electrooculography, electrocardiography, oxygen saturation, snoring, oral and nasal airflow, thoracic and abdominal respiratory effort, and body position were monitored. PSG provided comprehensive measures such as total sleep time (TST), sleep efficiency, sleep onset latency, wake after sleep onset (WASO), rapid eye movement (REM) latency, sleep stage distribution, apnea–hypopnea index (AHI), oxygen desaturation index (ODI), mean and nadir oxygen saturation, time spent with oxygen saturation below 90% (T90%), arousal index, periodic limb movement (PLM) index, and heart rate. Sleep-disordered breathing events were further categorized based on body position (supine versus non-supine) and sleep stage REM versus non-REM). Sleep stages and respiratory events were scored in accordance with the latest international guidelines [15, 16]. Apneas were defined as a ≥ 90% reduction in airflow persisting for at least 10 s, while hypopneas were identified as a ≥ 30% reduction in airflow lasting ≥ 10 s and associated with either a ≥ 3% drop in oxygen saturation or an arousal. OSA was considered present when the AHI was ≥ 5 events per hour, with moderate-to-severe OSA defined as AHI ≥ 15 events/hour and severe OSA as AHI ≥ 30 events/hour [17].

### Insomnia assessment

Insomnia symptoms were evaluated using questions addressing difficulty initiating sleep, self-reported sleep latency of 30 min or more, trouble maintaining sleep, or use of hypnotic medications. Participants reporting “often” or “very often” for at least one of these items were categorized as having insomnia.

### Diabetes mellitus assessment

The diagnosis of DM was maintained based on the self-reported physician diagnosis as well as the reported anti-diabetic medications. Data regarding diabetes type, disease duration, glycemic control, and diabetes-related complications were not available in the present analysis.

### Statistical analysis

All statistical analyses were conducted using IBM SPSS Statistics version 28.0 for Windows. Continuous variables were presented as mean ± standard deviation (SD) or, when appropriate, as median with interquartile range (25th–75th percentiles). Categorical variables were reported as frequencies and percentages. Normality of continuous data was assessed using the Shapiro–Wilk test. Comparisons between groups were performed using the Student’s t-test for continuous variables and the Chi-square test for categorical variables. A two-sided p-value of < 0.05 was considered statistically significant. Associations between COMISA and DM were evaluated using multivariable logistic regression, with results expressed as odds ratios (OR) and 95% confidence intervals (CI).

## Results

### Study population

**Fig. 1:**
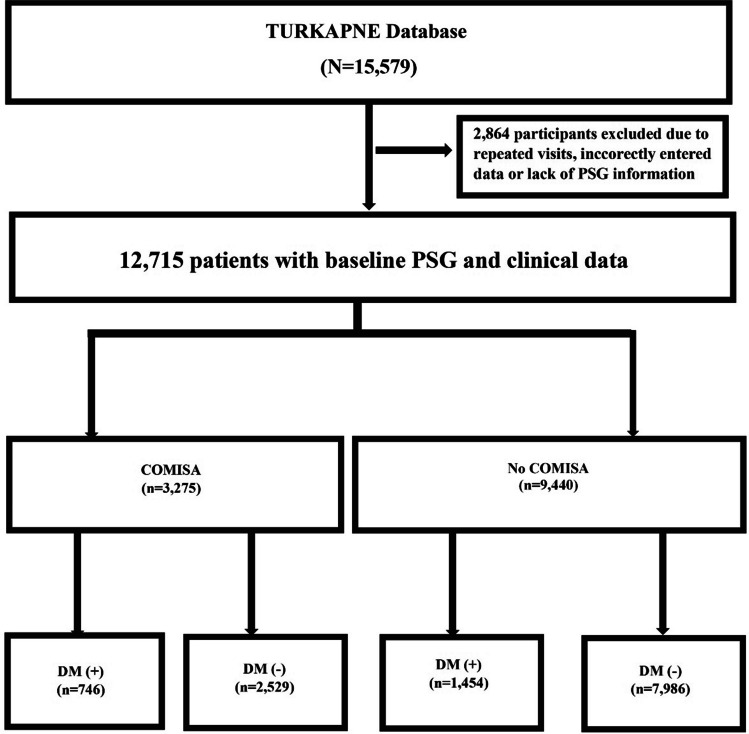
Flow chart of the study cohort. *COMISA* comorbid insomnia and sleep apnea, *DM* diabetes mellitus, *PSG* polysomnography, *TURKAPNE* Turkish sleep apnea database

A total of 12,715 patients with available baseline polysomnography and clinical data from the TURKAPNE cohort were included in the analysis (Fig. 1). The mean age of the cohort was 50.2 ± 12.1 years, 68.5% were male, and the mean BMI was 31.7 ± 6.1 kg/m^2^. Only a small proportion of patients (*n* = 153) were classified as having insomnia based solely on hypnotic medication use.

Overall, 3,275 patients (25.7%) had COMISA. DM was present in 2,200 participants (17.3%) in the entire cohort. The prevalence of DM was significantly higher among patients with COMISA compared with those without COMISA (22.8% vs. 15.4%, *p* < 0.001).

### Comparison between patients with and without diabetes mellitus

The comparison of demographic and clinical characteristics between patients with and without DM is presented in Table 1. Patients with DM were significantly older and had higher BMI values compared with non-diabetic individuals. The prevalence of obesity was also markedly higher in the diabetic group (73.3% vs. 52.2%, *p* < 0.001).

**Table 1:** The comparison of demographical and clinical features in all patients with and without diabetes mellitus

	DM (+) (*n* = 2200)	DM (-) (*n* = 10515)	*P* value
Age (years) (mean ± SD)	55.5 ± 10.5	49.0 ± 12.1	< 0.001
Female/male (*n*)	931/1269	3070/7445	< 0.001
BMI (kg/m2)	34.3 ± 6.5	31.1 ± 5.8	< 0.001
Obesity (BMI > 30 kg/m2) (%)	73.3	52.2	< 0.001
Current smoking (%)	27.2	33.9	< 0.001
Married (%)	85.9	83.5	0.005
ESS > 10 (%)	36.1	25.7	< 0.001
No education (%)	8.3	6.7	< 0.001
Primary school (%)	34.8	25.4	
Secondary school (%)	12.0	11.6	
High school (%)	24.3	26.4	
University (%)	20.6	29.9	
Neither insomnia nor OSA (*n*, %)	73 (3.3)	837 (8.0)	< 0.001
Insomnia only (*n*, %)	54 (2.5)	334 (3.2)	
OSA only (*n*, %)	1327 (60.3)	6815 (64.8)	
COMISA (*n*, %)	746 (33.9)	2529 (24.1)	
Hypertension (*n*, %)	1538 (69.9)	2460 (23.4)	< 0.001
Coronary artery disease (*n*, %)	743 (33.8)	2529 (11.5)	< 0.001
Obstructive airway disease (*n*, %)	500 (22.7)	1046 (9.9)	< 0.001
Psychiatric diseases (*n*, %)	238 (10.8)	557 (5.3)	< 0.001

*BMI* body mass index, *COMISA* comorbid obstructive sleep apnea and insomnia, *DM* diabetes mellitus, *ESS* epworth sleepiness score, *OSA *obstructive sleep apnea

As shown in Table 2, diabetic patients showed higher rates of excessive daytime sleepiness and several comorbid conditions, including hypertension, coronary artery disease, obstructive airway disease, and psychiatric disorders (all *p* < 0.001). Smoking was less frequent among patients with DM (27.2% vs. 33.9%, *p* < 0.001). Difficulty maintaining sleep was more prevalent than difficulty initiating sleep among patients with diabetes within the COMISA subgroup, suggesting a potential link between metabolic dysfunction and sleep maintenance insomnia.

**Table 2:** The comparison of demographical and clinical factors in COMISA patients with and without diabetes mellitus (*n* = 3275)

	COMISA (+) DM (+) (*n* = 746)	COMISA (+) DM (-) (*n* = 2529)	*p* value
Age (years) (mean ± SD)	56.1 ± 10.6	50.8 ± 12.1	< 0.001
BMI (kg/m2) (mean ± SD)	34.6 ± 6.6	31.8 ± 5.9	< 0.001
Female/male (n)	390/356	921/1608	< 0.001
Obesity (BMI > = 30 kg/m2) (%)	73.3	57.5	< 0.001
Current smoker (%)	26.4	35.9	< 0.001
Married (%)	81.2	81.1	0.954
EDS > 10 (%)	34.0	27.8	< 0.001
No education (%)	10.3	7.9	< 0.001
Primary school (%)	35.0	28.9	
Secondary school (%)	11.0	10.3	
High school (%)	25.3	26.2	
University (%)	18.4	26.7	
Hypnotics use (%)	11.1	9.9	0.324
Total sleep time < 6 h (%)	32.1	34.8	0.181
Difficulty in falling asleep (%)	58.5	52.9	0.009
Difficulty in maintaining sleep (%)	50.8	44.0	0.002
Subjective sleep latency ≥ 30 min (%)	58.9	63.4	0.027
Hypertension (%)	73.6	28.8	< 0.001
Cardiac Disease (%)	44.6	18.0	< 0.001
Airway Disease (%)	27.3	12.8	< 0.001
Hypothyroidism (%)	10.6	4.5	< 0.001
Psychiatric Disease (%)	13.0	8.1	< 0.001

*BMI* body mass index, *DM* diabetes mellitus, *EDS* excessive daytime sleepiness

Sleep disorder distribution differed significantly between the two groups. COMISA was more prevalent in patients with DM compared with those without DM (33.9% vs. 24.1%, *p* < 0.001).

### Factors associated with COMISA

Multivariable logistic regression analysis was performed to identify factors independently associated with COMISA. After adjustment for age, sex, BMI, smoking status, education level, and comorbidities, DM remained significantly associated with COMISA (adjusted OR 1.17; 95% CI 1.05–1.31; *p* = 0.006).

**Fig. 2:**
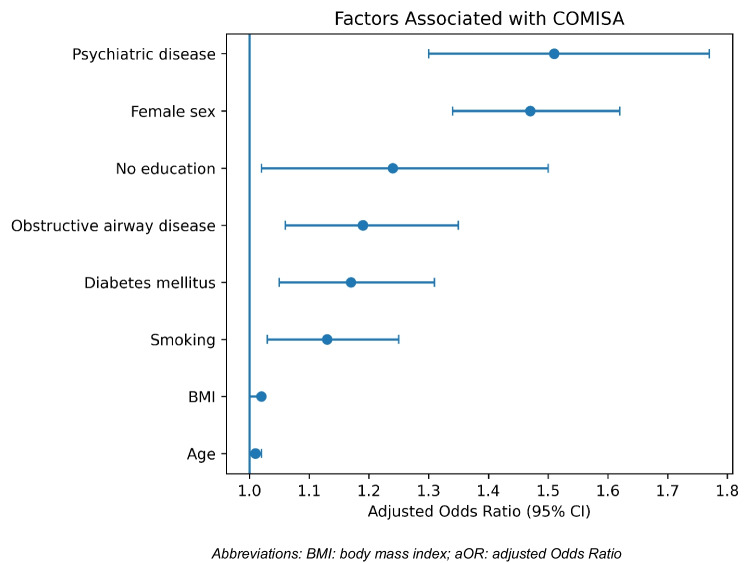
Factors associated with COMISA in multivariable logistic regression analysis. Points represent adjusted odds ratios (aOR) and horizontal bars indicate 95% confidence intervals. The vertical reference line represents an odds ratio of 1. *BMI* body-mass-index, comorbid insomnia and sleep apnea, *OAD* obstructive airway disease

Female sex (aOR 1.47; 95% CI 1.34–1.62), psychiatric disease (aOR 1.51; 95% CI 1.30–1.77), obstructive airway disease (aOR 1.19; 95% CI 1.06–1.35), smoking (aOR 1.13; 95% CI 1.03–1.25), lower education level (aOR 1.24; 95% CI 1.02–1.50), and higher BMI were also independently associated with COMISA (Fig. 2). The multivariable model was adjusted for age, sex, BMI, smoking status, education level, and comorbidities.

### Characteristics of COMISA patients with and without diabetes mellitus

Among the 3,275 patients with COMISA, 746 (22.8%) had DM. COMISA patients with DM were significantly older and had higher BMI values compared with those without DM. Female sex and obesity were more common in the diabetic group (*p* < 0.001 for both).

In addition, diabetic patients reported more frequent insomnia symptoms, including difficulty initiating sleep (58.5% vs. 52.9%, *p* = 0.009) and difficulty maintaining sleep (50.8% vs. 44.0%, *p* = 0.002). Excessive daytime sleepiness and several comorbid conditions, including hypertension, cardiac disease, obstructive airway disease, hypothyroidism, and psychiatric disorders, were also significantly more prevalent among diabetic individuals (all *p* < 0.001).

### Polysomnographic characteristics

Polysomnographic comparisons between COMISA patients with and without DM are shown in Table 3. Diabetic patients exhibited significantly greater impairment in sleep architecture and respiratory parameters.

**Table 3:** Polysomnographic features of COMISA patients with and without diabetes mellitus

	COMISA (+) DM (+) (*n* = 746)	COMISA (+) DM (-) (*n* = 2579)	*P* value
TST (min)	353.5 (297.6–402.2)	369.0 (317.5–414.3)	< 0.001
WASO (min)	50.2 (23.1–87.7)	37.8 (18.0–71.0)	< 0.001
Sleep efficiency (%)	86.2 (75.4–94.2)	87.6 (77.6–94.1)	0.069
N1 (%)	4.5 (1.3–10.2)	3.4 (0.6–8.0)	< 0.001
N2 (%)	58.2 (47.7–70.9)	59.9 (48.9–69.9)	0.625
N3 (%)	20.8 (12.7–29.8)	20.8 (13.1–30.0)	0.545
REM (%)	12.3 (7.4–18.1)	13.4 (8.3–18.5)	0.018
AHI (events/hr)	30.0 (16.0–55.0)	24.0 (13.0–46.1)	< 0.001
ODI (events/hr)	26.0 (13.0–52.6)	20.0 (9.8–42.0)	< 0.001
Time spent SpO_2_ < 90% (min)	18.6 (3.0–78.0)	11.9 (1.7–67.0)	0.001
Nadir SpO_2_ (%)	81.0 (72.0–85.0)	82.0 (75.0–86.3)	< 0.001
Arousal index	18.1 (8.0–34.0)	14.8 (6.2–30.2)	< 0.001
PLM index	8.6 (2.4–25.1)	7.3 (2.2–21.7)	0.068

Continuous data are presented as median and 25%−75% quartiles. Categorical data are presented as percentage

*AHI* apnea–hypopnea index, *COMISA* comorbid insomnia and sleep apnea, *DM* diabetes mellitus, *N1* non-rapid eye movement sleep stage 1, *N2* non-rapid eye movement sleep stage 2, *N3* non-rapid eye movement sleep stage 3, *ODI* oxygen desaturation index, *PLM* periodic limb movement, *REM* rapid eyemovement sleep, *SpO2* oxygen saturation by pulse oximeter, *TST* total sleep time, *WASO* wake after sleep onset

Specifically, diabetic individuals had shorter TST (353.5 vs. 369.0 min, *p* < 0.001) and increased WASO (50.2 vs. 37.8 min, *p* < 0.001). Sleep fragmentation was also reflected by a higher arousal index (18.1 vs. 14.8 events/hour, *p* < 0.001).

Regarding sleep stages, diabetic patients spent more time in light N1 sleep and less time in REM sleep compared with non-diabetic individuals (*p* < 0.001 and *p* = 0.018, respectively), while N2 and N3 sleep proportions were similar between groups.

Respiratory disturbance was significantly more severe in the diabetic group, with higher AHI (30.0 vs. 24.0 events/hour) and ODI (26.0 vs. 20.0 events/hour) (both *p* < 0.001). Additionally, diabetic patients spent more T90% (18.6 vs. 11.9 min, *p* = 0.001) and exhibited lower nadir oxygen saturation levels (81.0% vs. 82.0%, *p* < 0.001).

## Discussion

### Main findings

This large cross-sectional analysis of the TURKAPNE cohort demonstrated that DM was independently associated with a greater likelihood of COMISA. In addition, among patients with COMISA, those with DM also demonstrated significantly more marked disturbances in both sleep architecture and sleep-disordered breathing indices. Shorter TST, greater sleep fragmentation, and higher indices of respiratory disturbance were significant in patients with DM compared to the participants without DM. Thus, these findings highlight the substantial sleep-related burden in patients with DM and suggest that the coexistence of insomnia and OSA may define a clinically relevant phenotype associated with greater metabolic vulnerability.

### Relationship between COMISA and metabolic dysfunction

The connection between sleep disorders and metabolic disease is increasingly understood as bidirectional. Obstructive sleep apnea and insomnia have been both associated with impaired glucose metabolism and a higher risk of type 2 diabetes [4, 7]. OSA promotes metabolic dysregulation through intermittent hypoxia, sympathetic activation, systemic inflammation, and oxidative stress [4, 5]. Insomnia, on the other hand, has been associated with dysregulation of the hypothalamic–pituitary–adrenal axis activity and persistent hyperarousal, both of which may negatively influence insulin sensitivity and glucose homeostasis [6].

When insomnia and OSA co-exist, these pathways may reinforce on another. The repeated hypoxic stress caused by OSA may be intensified as a result of the physiological hyperarousal characteristic of insomnia, potentially worsening oxidative stress and inflammatory responses that contribute to insulin resistance and β-cell dysfunction [18–21]. Our results are in line with this context, as diabetic patients in the COMISA group had significantly greater respiratory disturbance and more nocturnal hypoxemia than non-diabetic patients.

### Impact of COMISA on sleep architecture

Another important observation in our study was the deterioration of sleep architecture among diabetic patients with COMISA [22–24]. These patients had shorter TST, increased WASO, and higher arousal indices, reflecting substantial sleep fragmentation. Additionally, a shift toward lighter sleep stages and reduced REM sleep was observed in diabetic individuals. Although the difference in nadir oxygen saturation reached statistical significance, its clinical relevance may be limited. Measures reflecting cumulative hypoxic burden, such as time spent with oxygen saturation below 90%, may provide a more clinically meaningful assessment.

Sleep fragmentation and reductions in restorative sleep stages have been shown to negatively affect glucose metabolism and insulin sensitivity [20]. Experimental studies demonstrate that even partial sleep deprivation or repeated nocturnal arousals can impair glucose tolerance and alter hormonal regulation of appetite and metabolism. Therefore, the disruption of sleep continuity observed in COMISA patients with DM may represent an additional pathway linking sleep disorders with metabolic dysfunction.

### Role of shared risk factors

Obesity represents one of the most important shared risk factors linking OSA, insomnia, and DM. Excess adiposity promotes upper airway collapsibility, systemic inflammation, and metabolic dysregulation, thereby contributing to both sleep-disordered breathing and impaired glucose metabolism [18]. Consistent with this relationship, obesity was highly prevalent in both the DM and COMISA groups in our cohort.

However, the association between DM and COMISA persisted even after adjustment for BMI and other confounding factors in the multivariable regression model. This suggests that mechanisms beyond obesity alone may contribute to the observed relationship. For instance, chronic inflammation, oxidative stress, and autonomic dysregulation, may represent overlapping biological pathways that contribute to both metabolic disease and sleep disturbances [20, 21].

Beyond obesity and traditional cardiometabolic comorbidities, the association between DM and COMISA may also reflect diabetes-specific mechanisms that directly impair sleep continuity and worsen nocturnal respiratory instability. Chronic hyperglycemia has been linked to autonomic dysfunction, peripheral neuropathy, nocturia, and altered thermoregulation, all of which may contribute to difficulty maintaining sleep and increased nocturnal arousals. At the same time, recurrent sleep fragmentation and intermittent hypoxemia may aggravate insulin resistance and glycemic dysregulation, creating a self-perpetuating cycle between metabolic dysfunction and disturbed sleep. From this perspective, COMISA in individuals with DM may represent not merely the coexistence of 2 common disorders, but a more vulnerable clinical phenotype characterized by mutually reinforcing metabolic and sleep-related abnormalities. This interpretation may help explain why diabetic patients in our cohort exhibited both more severe insomnia-related nocturnal disruption and a greater burden of sleep-disordered breathing.

The association between diabetes and COMISA may also reflect a bidirectional relationship. Diabetes-related factors such as nocturia, medication effects, and autonomic dysfunction may contribute to sleep disruption, particularly difficulties in maintaining sleep. In line with this, difficulty maintaining sleep was more prevalent than difficulty initiating sleep among diabetic patients in our cohort, suggesting that sleep maintenance insomnia may be more closely linked to metabolic dysfunction.

### Sex differences and clinical phenotypes

Our findings also suggest potential sex-related differences in the COMISA phenotype. Within the COMISA subgroup, diabetic patients were more frequently female. Previous research indicates that women with OSA often present with atypical symptoms, including insomnia complaints, fatigue, or mood disturbances, which may contribute to underdiagnosis of sleep-disordered breathing in female populations [25, 26] Hormonal changes related to menopause, including declines in progesterone and estrogen, may contribute to both increased upper airway collapsibility and higher prevalence of insomnia symptoms, potentially explaining the observed sex differences.

In addition, insomnia symptoms are more common in women and are often accompanied by psychiatric comorbidities such as anxiety and depression. These factors may interact with the psychological and physiological burden of DM, potentially leading to a more complex clinical phenotype in which metabolic disease, sleep disruption, and emotional dysregulation occur together.

### Clinical implications

The coexistence of insomnia and OSA is increasingly recognized as a distinct clinical phenotype that may carry greater health consequences than either condition alone. Our findings suggest that patients with DM may be particularly susceptible, with COMISA in this group being associated with more severe sleep fragmentation and respiratory disturbances.

These results highlight the importance of routinely screening individuals with DM for both insomnia symptoms and sleep-disordered breathing. Identifying COMISA early could help clinicians adopt integrated treatment strategies, such as positive airway pressure therapy for OSA together with cognitive behavioral therapy for insomnia. Such combined approaches may improve sleep quality and potentially reduce cardiometabolic risk.

### Strengths and limitations

The main strengths of this study include the large sample size, the nationwide multicenter design, and the use of full-night polysomnography to objectively characterize sleep parameters. However, several limitations should be acknowledged. First, DM and other comorbidities were identified based on self-reported physician diagnosis and medication use, which may introduce reporting bias. Second, insomnia was assessed using symptom-based questions rather than standardized diagnostic criteria such as the International Classification of Sleep Disorders or validated questionnaires (e.g., Insomnia Severity Index), which may have led to misclassification. Third, diabetes-related information such as glycemic control, disease duration, and treatment details was not available, limiting further phenotypic characterization. Fourth, information on the temporal sequence between OSA, insomnia symptoms, and diabetes diagnosis was not available in the present dataset. Fifth, although hypnotic medication use may reflect factors beyond insomnia itself, only a small proportion of patients in our cohort were classified solely based on medication use, suggesting that its impact on the overall findings is likely limited. Moreover, detailed information on specific antidiabetic medication classes, including GLP-1 receptor agonists, was not available. However, given the relatively recent introduction of these agents in Türkiye, their impact on diabetes classification in this cohort is likely to be limited. Additionally, because of the cross-sectional design of the study, conclusions regarding causality or temporal relationships between COMISA and DM could not be stated. Lastly, the study population was derived from a Turkish cohort, which may limit the generalizability of the findings to other populations with different demographic and clinical characteristics.

## Conclusions

In this large nationwide cohort, diabetes mellitus was associated with a higher prevalence of COMISA. Moreover, diabetic patients with COMISA exhibited more severe sleep fragmentation and greater respiratory disturbance compared with non-diabetic individuals. These findings support the view that COMISA represents a clinically important sleep disorder phenotype in patients with diabetes and reinforce the need for integrated screening and management of sleep disorders in this population.

## Data Availability

Data collected for the study, including de-identified individual participant data will be made available to others within 6 months after the publication of this article, as will additional related documents (study protocol, statistical analysis plan, and informed consent form), for academic purposes (e.g., meta-analyses), upon request to the senior author (yuksel.peker@lungall.gu.se), and with a signed data access agreement.

## Declarations

### Generative AI and AI-assisted technologies in the writing process

During the preparation of this work the author(s) used ChatGPT 5 in order to improve language and readability. After using this tool/service, the author(s) reviewed and edited the content as needed and take(s) full responsibility for the content of the publication.

### Clinical trial registration

NCT02784977.

## Abbreviations

*AHI*: Apnea–hypopnea index
*BMI*: Body mass index
*CI*: Confidence interval
*COMISA*: Comorbid insomnia and sleep apnea
*COPD*: Chronic obstructive pulmonary disease
*DM*: Diabetes mellitus
*ESS*: Epworth sleepiness scale
*N1*: Non-rapid eye movement sleep stage 1
*N2*: Non-rapid eye movement sleep stage 2
*N3*: Non-rapid eye movement sleep stage 3
*ODI*: Oxygen desaturation index
*OR*: Odds ratio
*OSA*: Obstructive sleep apnea
*PAP*: Positive airway pressure
*PLM*: Periodic limb movement
*PSG*: Polysomnography
*REM*: Rapid eye movement
*SD*: Standard deviation
*T90%*: Time spent with oxygen saturation below 90%
*TST*: Total sleep time
*WASO*: Wake after sleep onset
